# KIF14–AKT axis regulates ferroptosis sensitivity in triple-negative breast cancer

**DOI:** 10.1515/biol-2025-1324

**Published:** 2026-05-04

**Authors:** Chunyu Shi, Weiyi Fang, Yingqi Zhu, Ziying Xie, Ming Zhang

**Affiliations:** Yunkang School of Medicine and Health, Guangzhou Nanfang College, Guangzhou 510080, China

**Keywords:** triple-negative breast cancer, KIF14, ferroptosis, AKT, therapeutic target

## Abstract

Triple-negative breast cancer (TNBC) is an aggressive breast cancer subtype with limited targeted treatment options. Ferroptosis has emerged as a potential therapeutic vulnerability in TNBC, but its upstream regulation remains incompletely defined. Here, we combined bioinformatics analysis with cell-based experiments to investigate the role of kinesin family member 14 (KIF14) in ferroptosis sensitivity. KIF14 was upregulated in TNBC datasets and associated with poor prognosis. In MDA-MB-468 cells, KIF14 knockdown reduced cell viability, increased malondialdehyde and intracellular Fe^2+^ levels, and induced mitochondrial changes consistent with ferroptosis. These effects were partially reversed by ferrostatin-1. Comparison with inhibitors of apoptosis, necroptosis, and pyroptosis showed that only ferrostatin-1 produced a clear rescue effect, supporting a predominantly ferroptosis-associated phenotype. Mechanistically, KIF14 depletion reduced phosphorylation of AKT serine/threonine kinase (AKT) and altered ferroptosis-related proteins, including decreased glutathione peroxidase 4 (GPX4) and solute carrier family 7 member 11 (SLC7A11) and increased acyl-CoA synthetase long-chain family member 4 (ACSL4). The AKT activator SC79 partially reversed these biochemical and phenotypic changes. Reciprocal co-immunoprecipitation further supported an association between endogenous KIF14 and AKT. In addition, KIF14 knockdown had minimal effects on the viability of normal MCF-10A cells. Together, these findings support a functional link between KIF14, AKT signaling, and ferroptosis sensitivity in TNBC**.**

## 1 Introduction

Triple-negative breast cancer (TNBC) represents approximately 15–20 % of all breast cancer cases and is characterized by the absence of estrogen receptor (ER), progesterone receptor (PR), and human epidermal growth factor receptor 2 (HER2) expression [1], 2]. The lack of established hormonal or targeted therapeutic avenues relegates cytotoxic chemotherapy to the frontline of TNBC management [3]. However, resistance to chemotherapy is common, leading to early relapse and poor clinical outcomes, underscoring the urgent need for novel treatment strategies [4].

Ferroptosis has garnered substantial attention as a non-apoptotic cell death modality characterized by unrestrained iron-dependent lipid peroxidation [5]. Mounting evidence indicates that TNBC cells harbor intrinsic metabolic features – including elevated polyunsaturated fatty acid incorporation into membrane phospholipids and heightened iron uptake – that render them particularly susceptible to ferroptotic insults [6], 7]. However, the precise molecular gatekeepers that dictate ferroptosis sensitivity versus resistance in TNBC have yet to be comprehensively mapped [8].

Kinesin family member 14 (KIF14) is a kinesin family motor protein increasingly recognized as an oncogenic regulator in multiple cancers [9], 10]. Beyond its canonical mitotic functions, KIF14 has been reported to potentiate phosphoinositide 3-kinase (PI3K)/AKT signaling – a pathway notorious for its pleiotropic pro-survival effects in cancer cells [11], 12]. Prior investigations have linked KIF14 overexpression to tumor progression and treatment resistance in multiple cancers, including TNBC; however, whether KIF14 impinges on ferroptosis regulation has remained unexplored [11], 13].

Given the convergent relevance of both KIF14 and ferroptosis to TNBC biology, we hypothesized that KIF14 might modulate ferroptosis susceptibility through AKT serine/threonine kinase (AKT)-dependent mechanisms. In this study, we investigated the role of KIF14 in modulating ferroptosis sensitivity in TNBC cell lines using a combination of bioinformatics analysis, molecular biology techniques, and functional assays. Our findings reveal a previously unrecognized KIF14-AKT signaling axis that suppresses ferroptosis in TNBC cells, suggesting that targeting this pathway could sensitize TNBC to ferroptosis-inducing therapies.

## 2 Materials and methods

### 2.1 Data collection and bioinformatics analysis

Gene expression datasets GSE45827 and GSE65194 were obtained from the Gene Expression Omnibus (GEO) database. The GSE45827 dataset contained 41 triple-negative breast cancer (TNBC) samples and 11 normal breast tissue samples, while GSE65194 included 153 breast cancer samples with clinical annotation data. Metadata were parsed to classify samples – “triple-negative” or “basal-like” tumors and TNBC cell lines versus all others – mirroring the discovery cohort and maximizing statistical power. Raw expression data were processed using the limma package in R software (version 4.2.0). Differential expression analysis was performed using the empirical Bayes method with Benjamin–Hochberg false discovery rate correction. Genes with adjusted *p*-value < 0.05 and absolute log2 fold change > 2 were considered significantly differentially expressed, and KIF14 overexpression was externally validated in GSE65194 via the same criteria.

Ferroptosis-related genes were identified through comprehensive literature review and database mining from FerrDb (http://www.zhounan.org/ferrdb). Gene set enrichment analysis (GSEA) was conducted using the clusterProfiler package to evaluate ferroptosis pathway activation. Survival analysis was performed using The Cancer Genome Atlas (TCGA) breast cancer dataset through the Kaplan-Meier method with log-rank test. Protein expression validation was conducted using immunohistochemistry data from The Human Protein Atlas (HPA) database.

### 2.2 Cell culture and transfection

Human TNBC cell lines MDA-MB-231, MDA-MB-468, and MCF-10A were obtained from the American Type Culture Collection (ATCC). MDA-MB-231 and MDA-MB-468 cells were cultured in Dulbecco’s modified Eagle medium (DMEM) supplemented with 10 % fetal bovine serum and 1 % penicillin-streptomycin at 37 °C with 5 % CO_2_. MCF-10A cells were maintained in mammary epithelial complete medium according to ATCC recommendations. Cell line authentication was performed using short tandem repeat profiling, and mycoplasma contamination was routinely tested.

Small interfering RNA (siRNA) targeting KIF14 and negative control siRNA were synthesized by GenePharma (Shanghai, China). The siRNA sequences were as follows: KIF14-1 siRNA-Sense: 5′-GUU​GGC​UAG​AAU​UGG​GAA​ATT-3′, KIF14 siRNA-Antisense: 5′-UUU​CCC​AAU​UCU​AGC​CAA​CTT-3′, KIF14-2 siRNA-Sense: 5′-CUC​AGA​GCA​AGU​UGG​AUA​UTT-3′, KIF14 siRNA-Antisense: 5′-AUA​UCC​AAC​UUG​CUC​UGA​GTT-3′, and negative control: Sense: 5′-UUC​UCC​GAA​CGU​GUC​ACG​U TT-3′, Antisense: 5′-ACG​UGA​CAC​GUU​CGG​AGA​A TT-3′. Transfection was performed using Lipofectamine 3000 (Invitrogen) according to the manufacturer’s protocol. Cells were harvested 48 h post-transfection for subsequent experiments.

### 2.3 Quantitative real-time polymerase chain reaction (qRT-PCR) analysis

Total RNA was extracted using TRIzol reagent (Invitrogen) and reverse transcribed using the PrimeScript RT reagent kit (Takara). Quantitative real-time PCR was performed using SYBR Green Master Mix (Applied Biosystems) on a StepOnePlus Real-Time PCR System. The primer sequences were: KIF14 forward: 5′-CCT​CAC​CCA​CAG​TAG​CCG​A-3′, reverse: 5′-AAG​TGC​CAA​TCT​ACC​TAC​AGG​A-3′; GAPDH forward: 5′-GGA​GCG​AGA​TCC​CTC​CAA​AAT-3′, reverse: 5′-GGC​TGT​TGT​CAT​ACT​TCT​CAT​GG-3′. Relative expression levels were calculated using the 2^−ΔΔCt^ method with GAPDH as the internal control.

### 2.4 Cell viability and ferroptosis induction assays

Cell viability was assessed using the Cell Counting Kit-8 (CCK-8) assay (Dojindo). Cells were seeded in 96-well plates at a density of 5,000 cells per well and treated with various compounds. For rescue experiments, cells were pretreated with ferrostatin-1 (Fer-1, 2 μM, Sigma-Aldrich), Z-VAD-FMK (20 μM, Apoptosis Inhibitor, Promega), Nec-1 (10 μM, RIPK1 inhibitor, InvivoGen), VX-765 (10 μM, Caspase-1 inhibitor, R&D), or AKT activator SC79 (10 μM, Selleck Chemicals) for 2 h before siRNA transfection. CCK-8 reagent was added 48 h post-treatment, and absorbance was measured at 450 nm using a microplate reader.

### 2.5 Lipid peroxidation and iron detection

Malondialdehyde (MDA) levels were measured using a commercial MDA assay kit (Abcam) according to the manufacturer’s instructions. Briefly, cells were lysed in MDA lysis buffer, and the supernatant was collected after centrifugation. The MDA level was determined by measuring the absorbance at 532 nm and normalized to protein concentration. Intracellular ferrous iron (Fe^2+^) levels were detected using the FerroOrange fluorescent probe (Dojindo). Cells were incubated with 1 μM FerroOrange in serum-free medium for 30 min at 37 °C, washed with phosphate-buffered saline, and analyzed by fluorescence microscopy. Fluorescence intensity was quantified using ImageJ software.

### 2.6 Transmission electron microscopy

Cells were fixed with 2.5 % glutaraldehyde in phosphate buffer, post-fixed with 1 % osmium tetroxide, dehydrated through graded alcohols, and embedded in epoxy resin. Ultrathin sections were stained with uranyl acetate and lead citrate and examined using a transmission electron microscope. Mitochondrial morphology was evaluated qualitatively, and average mitochondrial area was quantified using ImageJ.

### 2.7 Western blot analysis

Protein extraction was performed using radioimmunoprecipitation assay (RIPA) lysis buffer containing protease and phosphatase inhibitors. Protein concentrations were determined using the bicinchoninic acid assay. Equal amounts of protein (30 μg) were separated by sodium dodecyl sulfate-polyacrylamide gel electrophoresis and transferred to polyvinylidene difluoride membranes. Membranes were blocked with 5 % non-fat milk and incubated overnight at 4 °C with primary antibodies against phosphorylated AKT (Ser473), glutathione peroxidase 4 (GPX4), solute carrier family 7 member 11 (SLC7A11), acyl-CoA synthetase long-chain family member 4 (ACSL4), and glyceraldehyde-3-phosphate dehydrogenase (GAPDH). After incubation with horseradish peroxidase-conjugated secondary antibodies, protein bands were visualized using enhanced chemiluminescence reagent and quantified using ImageJ.

### 2.8 Co-immunoprecipitation (Co-IP)

Endogenous KIF14–AKT complexes were isolated with the Dynabeads Protein G IP Kit (10007D, Thermo Fisher). MDA-MB-468 cells were lysed in ice-cold, non-denaturing buffer. Clarified lysates were pre-cleared with Protein G beads (30 min, 4 °C). Antibody–bead slurries were rotated with ∼500 µg lysate overnight at 4 °C, washed 4× in lysis/wash buffer, and eluted in 2× Laemmli at 95 °C for 5 min for SDS-PAGE/IB. Reciprocal IPs were performed, and 5–10 % input, IgG, and beads-only controls were run in parallel.

### 2.9 Statistical analysis

All experiments were performed in triplicate and repeated at least three times independently. Statistical analysis was conducted using GraphPad Prism 8.0 software. Data are presented as mean ± standard deviation. Student’s *t*-test was used for comparisons between two groups, and one-way analysis of variance followed by Tukey’s post-hoc test was used for multiple group comparisons. Survival analysis was performed using the Kaplan-Meier method with log-rank test. P-values less than 0.05 were considered statistically significant.

## 3 Results

### 3.1 KIF14 represents a critical differentially expressed gene affecting TNBC prognosis

**Figure 1:**
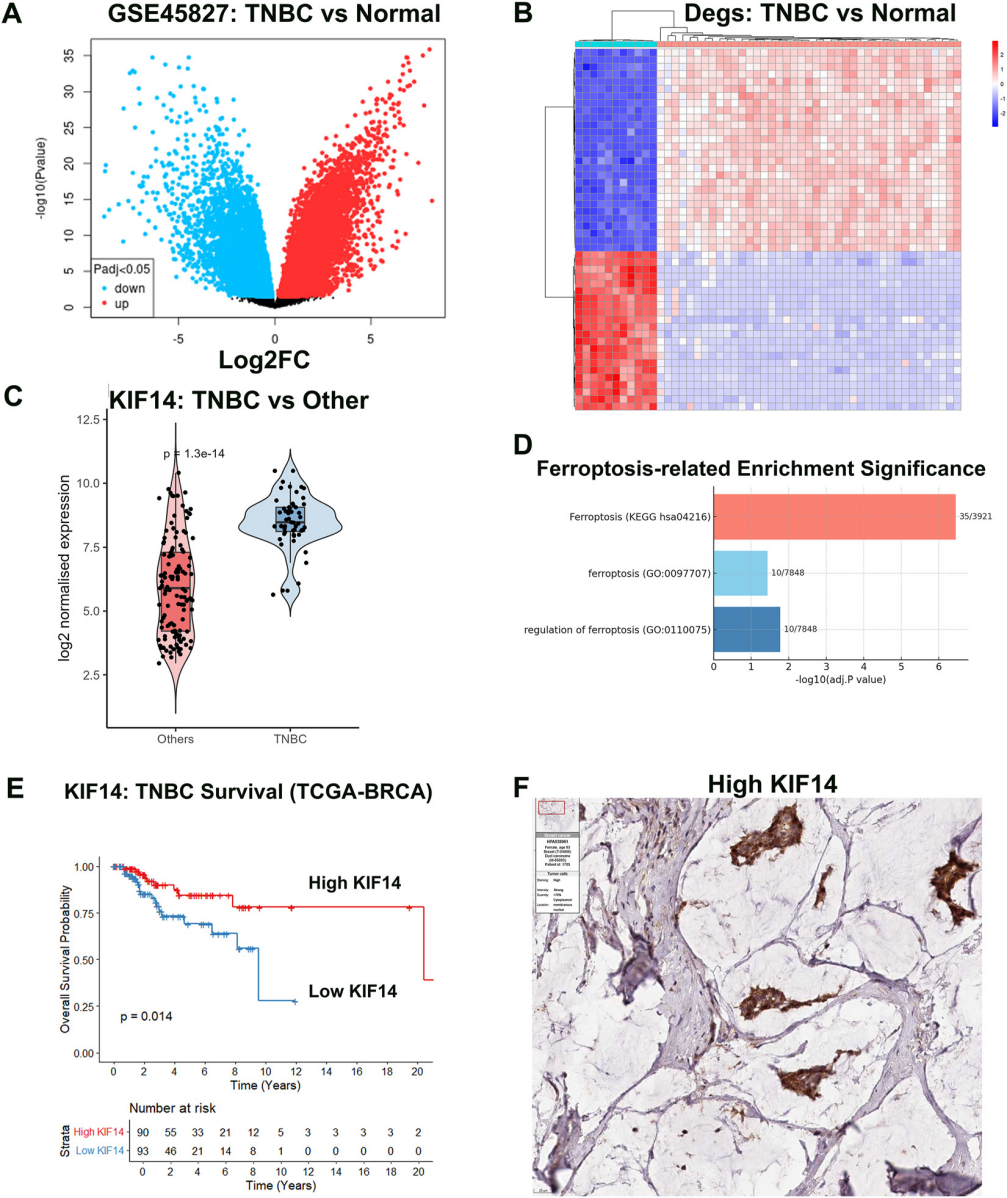
KIF14 represents a critical differentially expressed gene affecting TNBC prognosis. (A) Volcano plot of differential gene expression between TNBC and normal breast tissue (GSE45827). (B) Hierarchical clustering heatmap showing sample separation. (C) External validation of KIF14 overexpression in TNBC (GSE65194). ***P < 0.001. (D) Gene set enrichment analysis of ferroptosis-related pathways. (E) Kaplan–Meier survival curves from TCGA database. *P < 0.05. (F) Immunohistochemistry images showing KIF14 protein expression (HPA database).

To identify key molecular drivers in TNBC, we performed comprehensive bioinformatics analysis using publicly available datasets. Differential expression analysis of GSE45827 revealed 2,843 significantly dysregulated genes between TNBC and normal breast tissue samples. Among these, KIF14 emerged as a prominently upregulated gene with substantial fold change and statistical significance (Figure 1A). The volcano plot demonstrated KIF14’s position among the most significantly overexpressed genes in TNBC, with log2 fold change of 5.11 and adjusted *P*-value of 6.18 × 10^−24^. Unsupervised hierarchical clustering analysis successfully separated TNBC samples from normal controls, confirming the reliability of our sample classification and the robustness of the differential expression pattern (Figure 1B).

External validation using the independent GSE65194 dataset confirmed KIF14 upregulation in TNBC samples compared to normal breast tissue (*P* < 0.005), strengthening the evidence for consistent KIF14 dysregulation across different patient cohorts (Figure 1C). Gene set enrichment analysis revealed significant enrichment of ferroptosis-related pathways among the differentially expressed genes (*P* < 0.001), suggesting potential involvement of iron-dependent cell death mechanisms in TNBC pathogenesis (Figure 1D). Among the ferroptosis-associated genes, we identified 16 significantly dysregulated genes, including TNFAIP, TFR1, HMOX1, SLC7A11, followed by HMGCR, PCBP1, STEAP3, SAT1 and SLC3A2, indicating substantial perturbation of iron homeostasis and lipid metabolism pathways.

Survival analysis using The Cancer Genome Atlas breast cancer dataset demonstrated that patients with high KIF14 expression exhibited significantly shorter overall survival (hazard ratio = 1.68, 95 % confidence interval: 1.12–2.51, *P* < 0.05 compared to those with low KIF14 expression (Figure 1E). Protein expression validation through The Human Protein Atlas database confirmed KIF14 overexpression in breast cancer tissues compared to normal breast tissue, with strong nuclear and cytoplasmic staining patterns observed in malignant cells (Figure 1F).

### 3.2 KIF14 modulates ferroptosis sensitivity in TNBC cells

**Figure 2:**
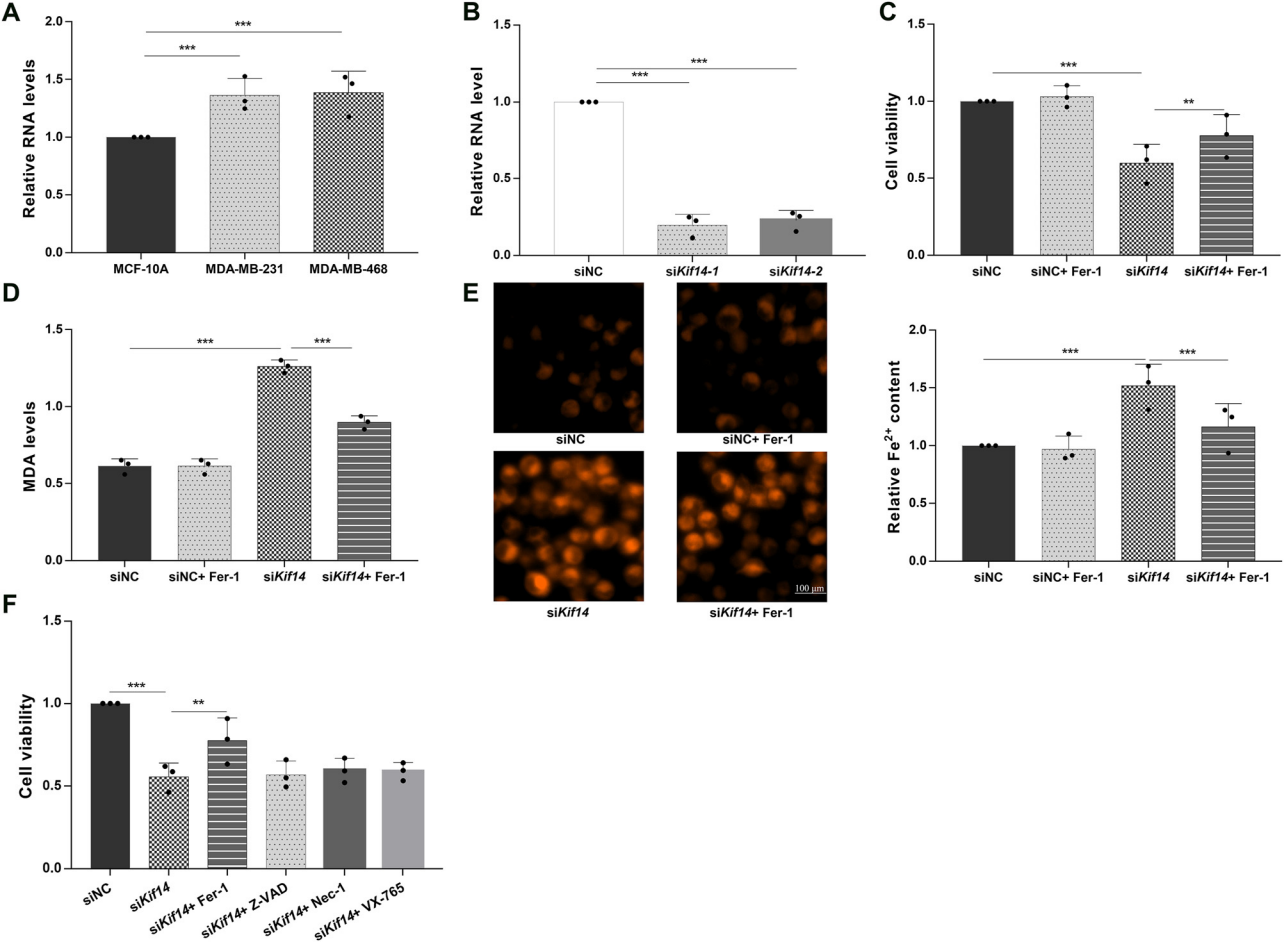
KIF14 modulates ferroptosis sensitivity in MDA-MB-468 cells. (A) KIF14 expression levels in MCF-10A, MDA-MB-231, and MDA-MB-468 cells measured by qRT-PCR. (B) qRT-PCR validation of KIF14 knockdown efficiency in MDA-MB-468 cells transfected with siNC, siKIF14-1, or siKIF14-2. (C) Cell viability in siNC, siNC + Fer-1, siKIF14, and siKIF14 + Fer-1 groups. (D) MDA levels in the indicated groups. (E) Representative FerroOrange images and quantification of intracellular Fe^2+^ levels. Scale bar = 100 μm. (F) Cell viability in siNC, siKIF14, siKIF14 + Fer-1, siKIF14 + Z-VAD-FMK, siKIF14 + Nec-1, and siKIF14 + VX-765 groups. Bars without significance brackets indicate no statistically significant difference relative to the siKIF14 group (*P* > 0.05). Data are presented as mean ± SD from three independent experiments. *P < 0.05, ***P < 0.001.

To investigate the functional role of KIF14 in TNBC cells, we first examined its expression in MCF-10A, MDA-MB-231, and MDA-MB-468 cells by qRT-PCR. KIF14 expression was highest in MDA-MB-468 cells, which were therefore selected for subsequent knockdown experiments (Figure 2A). To reduce the possibility of off-target effects, two independent siRNAs targeting KIF14 were used. qRT-PCR confirmed efficient reduction of KIF14 mRNA in MDA-MB-468 cells after transfection with either siKIF14-1 or siKIF14-2 (Figure 2B).

We next tested whether the loss of viability induced by KIF14 depletion was related to ferroptosis. In MDA-MB-468 cells, ferrostatin-1 (Fer-1) partially restored viability after KIF14 knockdown (Figure 2C). KIF14 depletion also increased malondialdehyde (MDA) levels and intracellular Fe^2+^ accumulation, and both changes were attenuated by Fer-1 treatment (Figure 2D and E). To further examine the specificity of the death phenotype, we compared the effects of inhibitors targeting different cell death pathways. Fer-1 partially rescued the loss of viability induced by KIF14 knockdown, whereas Z-VAD-FMK, Nec-1, and VX-765 did not produce a comparable rescue effect (Figure 2F). These findings support that KIF14 depletion predominantly induces a ferroptosis-associated death phenotype in TNBC cells.

### 3.3 KIF14 regulates TNBC cell ferroptosis sensitivity through AKT signaling

**Figure 3:**
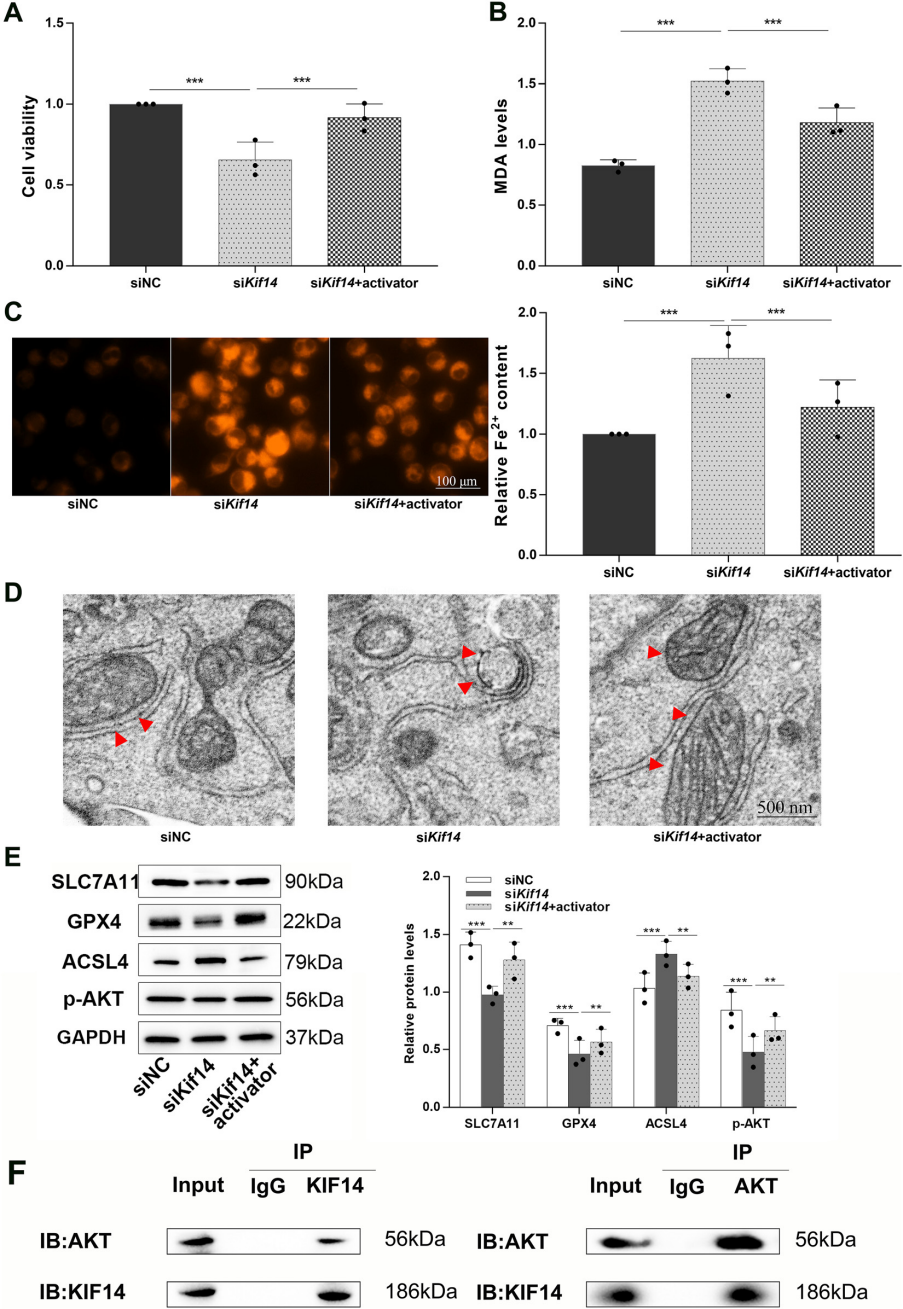
KIF14 regulates TNBC cell ferroptosis sensitivity through AKT signaling modulation. (A) Cell viability after KIF14 knockdown with or without SC79 treatment. (B) MDA levels in the indicated groups. (C) Representative FerroOrange images and quantification of intracellular Fe^2+^ levels. Scale bar = 100 μm. (D) Representative TEM images showing mitochondrial morphology in siNC, siKIF14, and siKIF14 + SC79 groups. Scale bar = 500 nm. (E) Western blot analysis of SLC7A11, GPX4, ACSL4, and p-AKT in the indicated groups, with GAPDH as the loading control. (F) Reciprocal co-immunoprecipitation showing the association between endogenous KIF14 and AKT in MDA-MB-468 cells. Data are mean ± SD (*n* = 3). **P < 0.01, ***P < 0.001.

To explore the mechanism underlying KIF14-dependent ferroptosis sensitivity, we examined the AKT pathway. KIF14 knockdown significantly reduced cell viability, whereas treatment with the AKT activator SC79 partially restored viability (Figure 3A). SC79 also reduced the increase in MDA and intracellular Fe^2+^ induced by KIF14 silencing (Figure 3B and C), indicating that AKT activation can counteract the ferroptosis-associated phenotype caused by KIF14 depletion.

TEM showed that KIF14 knockdown induced mitochondrial changes consistent with ferroptosis, including reduced mitochondrial size and condensed morphology. These changes were partially alleviated by SC79 treatment (Figure 3D). Quantification of average mitochondrial area further supported this observation (Supplementary Figure S2).

Western blot analysis showed that KIF14 depletion decreased p-AKT, GPX4, and SLC7A11 levels, while increasing ACSL4 expression. SC79 partially reversed these changes (Figure 3E). In addition, reciprocal co-immunoprecipitation assays showed that endogenous KIF14 and AKT were present in the same protein complex in MDA-MB-468 cells (Figure 3F). Together, these data support the involvement of AKT signaling in the ferroptosis-related effects observed after KIF14 knockdown.

### 3.4 KIF14 loss heightens ferroptotic vulnerability in MDA-MB-231

**Figure 4:**
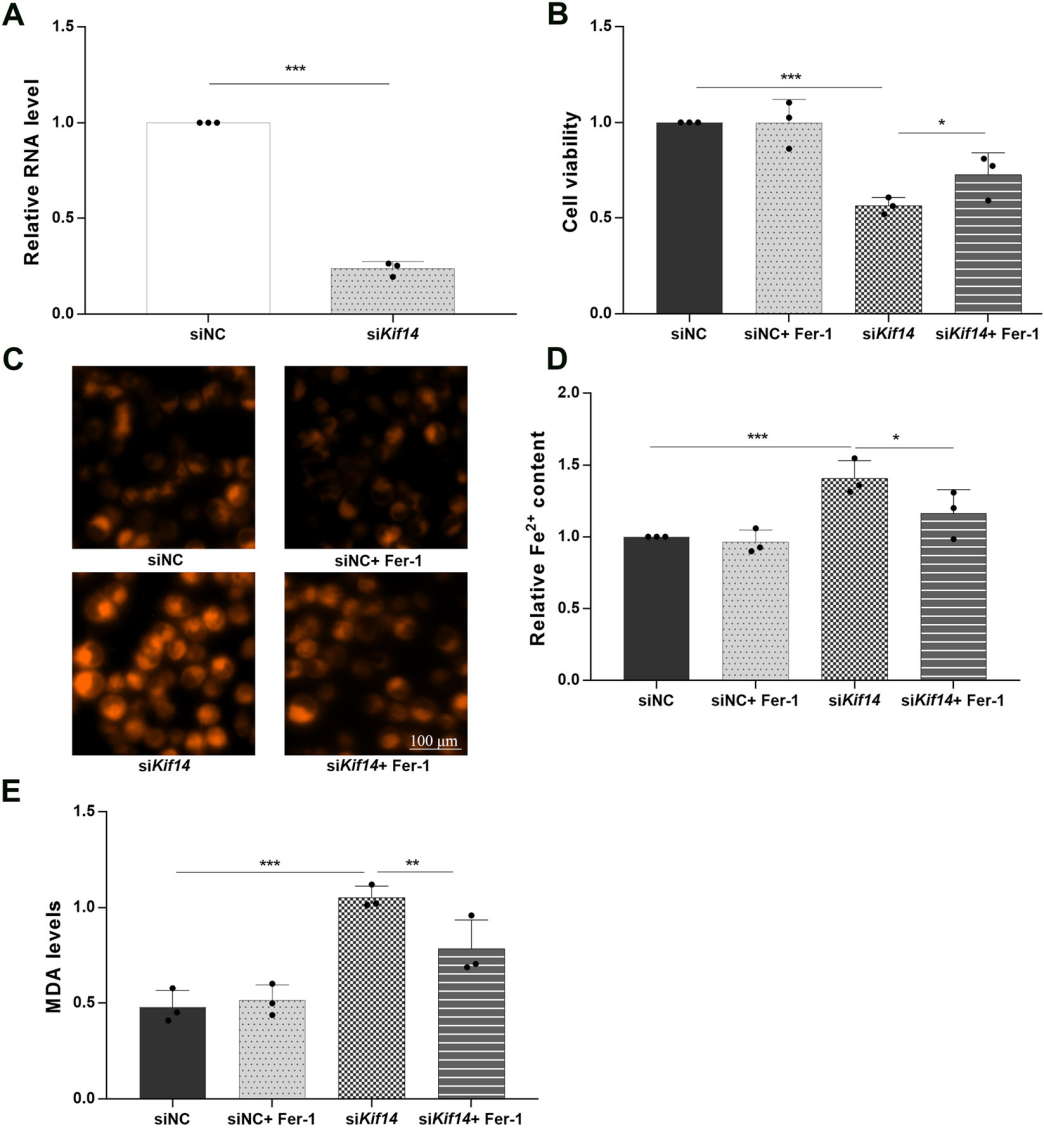
KIF14 loss increases ferroptosis and reduces viability in MDA-MB-231 cells, partially rescued by ferrostatin-1. (A) qPCR validation of KIF14 knockdown. (B) Cell viability in siNC, siNC + Fer-1, siKIF14, and siKIF14 + Fer-1 groups. (C) Representative fluorescence micrographs of probe staining (Methods) illustrating increased signal after KIF14 silencing and attenuation by Fer-1. (D) Relative intracellular Fe^2+^ content. (E) MDA levels. Data are mean ± SD (*n* = 3). ***P < 0.001; **P < 0.01; *P < 0.05.

KIF14 silencing was first confirmed in MDA-MB-231 cells, with qPCR showing a robust knockdown of KIF14 transcripts (Figure 4A). Functionally, KIF14 depletion significantly reduced cell viability relative to siNC controls, and co-treatment with ferrostatin-1 (Fer-1) partially restored viability (Figure 4B). In line with these changes, fluorescent probe imaging revealed stronger intracellular signals after KIF14 knockdown that were attenuated by Fer-1 (Figure 4C). Quantitatively, KIF14 loss increased labile Fe^2+^ levels and MDA content, whereas Fer-1 reduced both readouts (Figure 4D and E). *P < 0.05, **P < 0.01, ***P < 0.001.

## 4 Discussion

This study supports a functional link between KIF14, AKT signaling, and ferroptosis sensitivity in TNBC cells. KIF14 knockdown reduced viability, increased malondialdehyde and labile Fe2+ levels, and these effects were partially rescued by ferrostatin-1 in both MDA-MB-468 and MDA-MB-231 cells. In addition, comparison with inhibitors of apoptosis, necroptosis, and pyroptosis showed that only ferrostatin-1 produced a clear rescue effect. Accordingly, we interpret the phenotype as predominantly ferroptosis-associated rather than inferring that all cell death occurred exclusively through ferroptosis.

KIF14 is classically recognized as a mitotic kinesin involved in cytokinesis and tumor progression, and its overexpression has been reported across multiple malignancies [10], [14], [15], [16]. In TNBC and prostate cancer, KIF14 has also been linked to AKT phosphorylation and treatment resistance [11], 12]. Against this background, our data extend the biological role of KIF14 by connecting it to ferroptosis regulation, suggesting that KIF14 may couple oncogenic survival signaling to redox and lipid peroxidation defense in TNBC cells.

The rescue experiments with SC79 further support a functional contribution of AKT signaling to this process. KIF14 depletion reduced p-AKT levels and shifted ferroptosis-related proteins toward a pro-ferroptotic pattern, whereas AKT reactivation partially reversed the associated biochemical and phenotypic changes. This interpretation is consistent with growing evidence that PI3K/AKT/mTOR signaling restrains ferroptosis susceptibility in cancer; a recent review summarized this regulatory axis, and a TNBC study showed that TYMS knockdown promoted ferroptosis together with suppression of PI3K/AKT/mTOR signaling [17], 18]. Nevertheless, the present study does not define whether KIF14 regulates AKT directly or indirectly, nor whether AKT controls GPX4, SLC7A11, and ACSL4 mainly at transcriptional, translational, or post-translational levels in this setting.

Recent studies have revealed marked heterogeneity in ferroptosis susceptibility across TNBC subtypes, with GPX4-high tumors being particularly amenable to GPX4 inhibitor-induced ferroptosis [19]. Our identification of a KIF14-AKT-GPX4 link may therefore be especially relevant to TNBC subsets in which upstream survival signaling and ferroptosis defense are tightly coupled. In parallel, recent work has emphasized the bidirectional relationship between ferroptosis and the tumor immune microenvironment in TNBC and broader cancer immunity [20], 21]. In keeping with this framework, Yang et al. demonstrated that GPX4 inhibition enhanced antitumor immunity and improved the efficacy of anti-PD-1 therapy in preclinical TNBC models [19].

The relative sparing of MCF-10A cells after KIF14 knockdown further supports a potential therapeutic window. Malignant TNBC cells commonly display increased iron demand, lipid remodeling, and oxidative stress, features that can heighten ferroptosis dependency [22], 23]. Other recent TNBC studies likewise support the idea that upstream regulators can influence ferroptosis sensitivity and treatment response; for example, THEM6 enhanced carboplatin sensitivity by promoting ferroptosis through FDFT1 regulation [24]. Together, these findings support the view that KIF14 may function as an upstream vulnerability rather than merely a passive marker of aggressive disease.

From a translational perspective, our data suggest that KIF14 inhibition may sensitize selected TNBC cells to ferroptosis-inducing strategies and could potentially be explored in rational combinations with AKT-targeted agents or immunotherapy-oriented approaches. However, this possibility remains preclinical. Any biomarker-guided application involving KIF14, GPX4, SLC7A11, or ACSL4 will require validation in animal models, clinical specimens, and treatment-response datasets before therapeutic conclusions can be drawn [19], 23].

Several limitations should be noted. This study was performed mainly in cultured cells, so *in vivo* validation is still needed. In addition, although the co-immunoprecipitation and SC79 rescue results support involvement of AKT signaling, the precise mechanism linking KIF14 to AKT activation remains to be clarified.

## 5 Conclusions

In summary, this work unveils a previously unappreciated KIF14–AKT signaling axis that confers ferroptosis resistance in TNBC. These findings identify the KIF14–AKT axis as a potential regulator of ferroptosis sensitivity in TNBC and support further evaluation of this pathway as a therapeutic target.

## Supplementary Material

Supplementary Material
