# Supplementary material for: KIF14–AKT axis regulates ferroptosis sensitivity in triple-negative breast cancer
Source: Open Life Sci. 2026 May 4;21(1):20251324. doi: 10.1515/biol-2025-1324 (PMC13135662; doi:10.1515/biol-2025-1324)

A


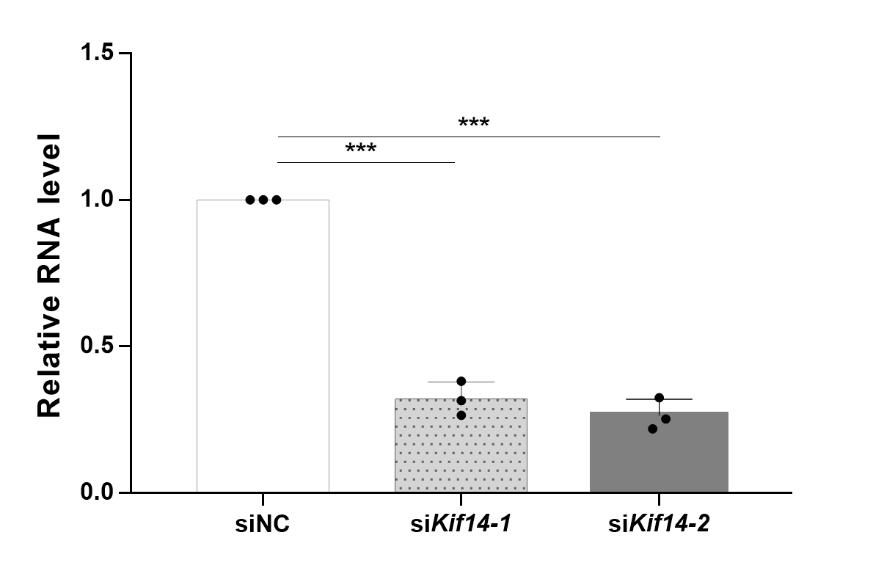


B


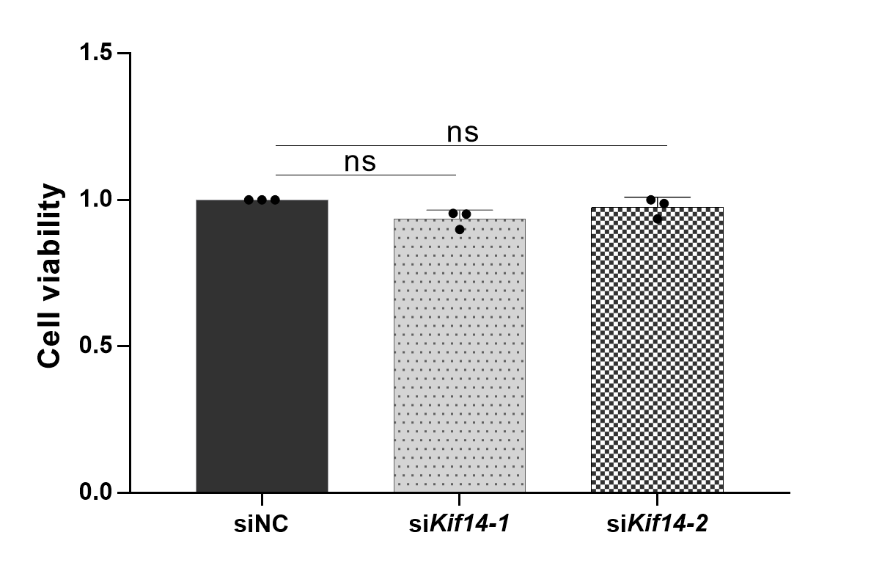


C

**Supplementary Figure S1. Validation of KIF14 knockdown in normal and TNBC cells.** (A) qRT-PCR analysis of KIF14 mRNA expression in MCF-10A cells transfected with siNC or siKIF14. (B) CCK-8 assay showing cell viability of MCF-10A cells after KIF14 knockdown. Data are presented as mean ± SD from three independent experiments. ns, not significant. ****P < 0.001.*


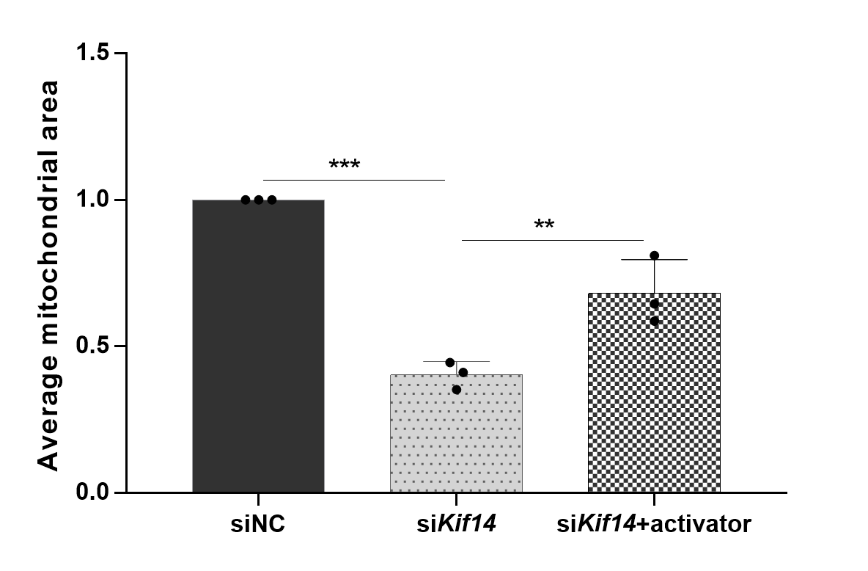


**Supplementary Figure S2. Quantification of average mitochondrial area in MDA-MB-468 cells.** Average mitochondrial area was significantly reduced after KIF14 knockdown and was partially restored by AKT activator treatment. Data are presented as mean ± SD. ***P < 0.01, ***P < 0.001.*

Western blot

SLC7A11


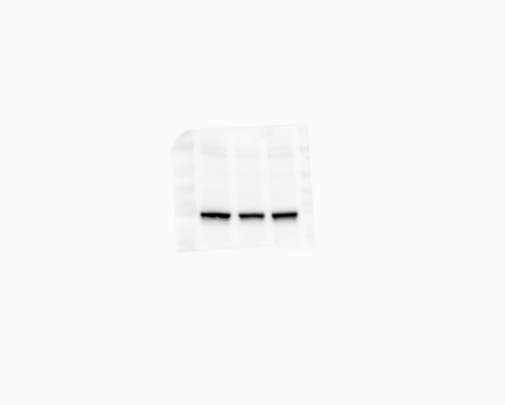

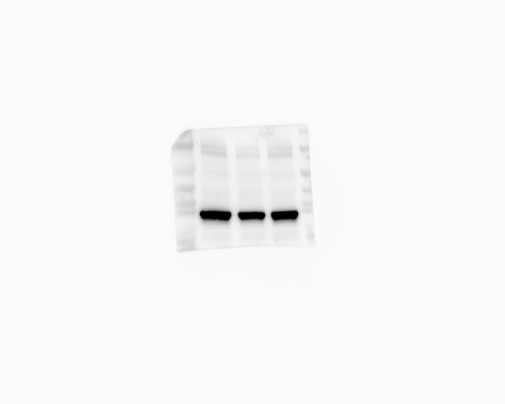


GPX4
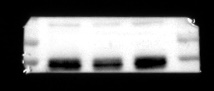

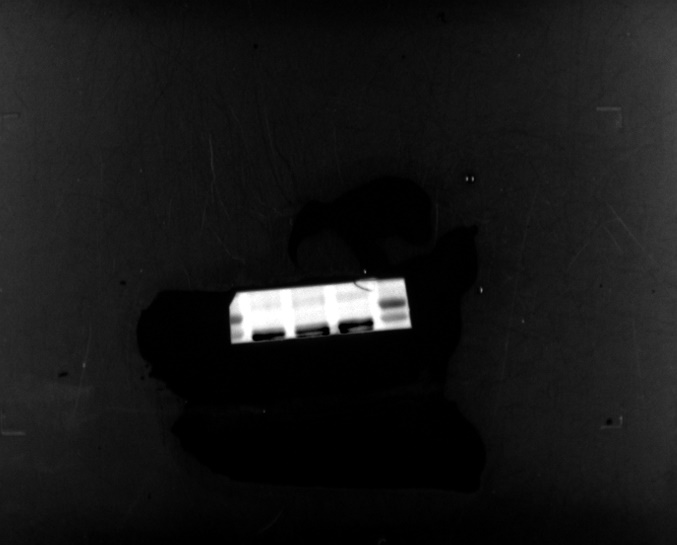




ACSL4


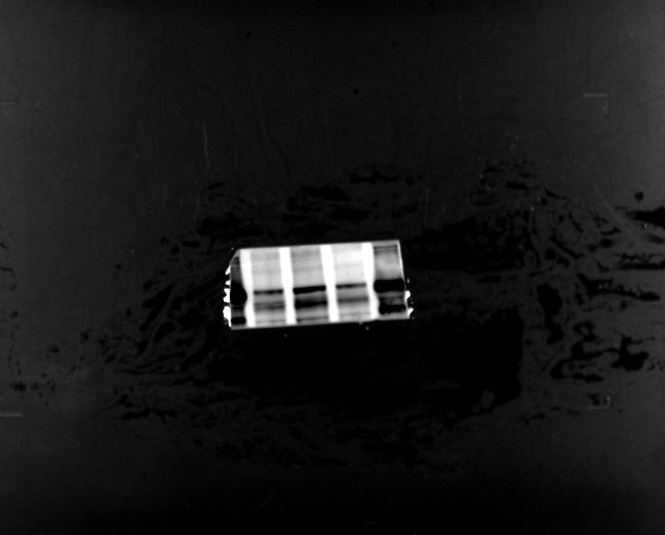

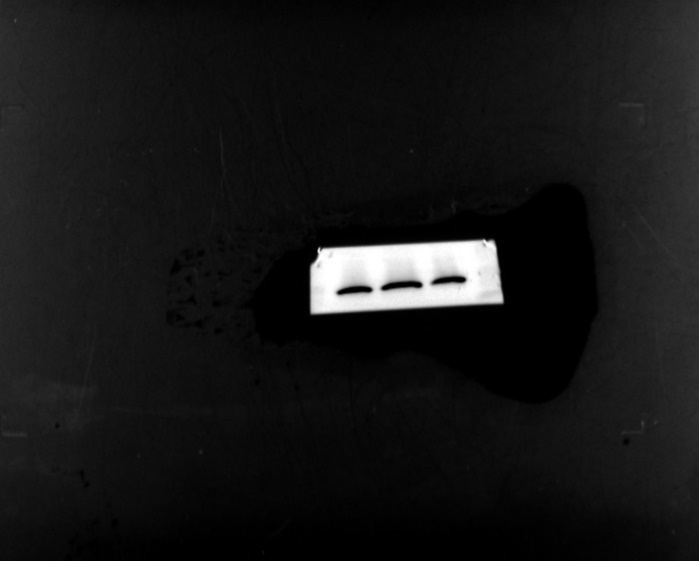


p-AKT
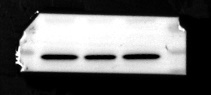

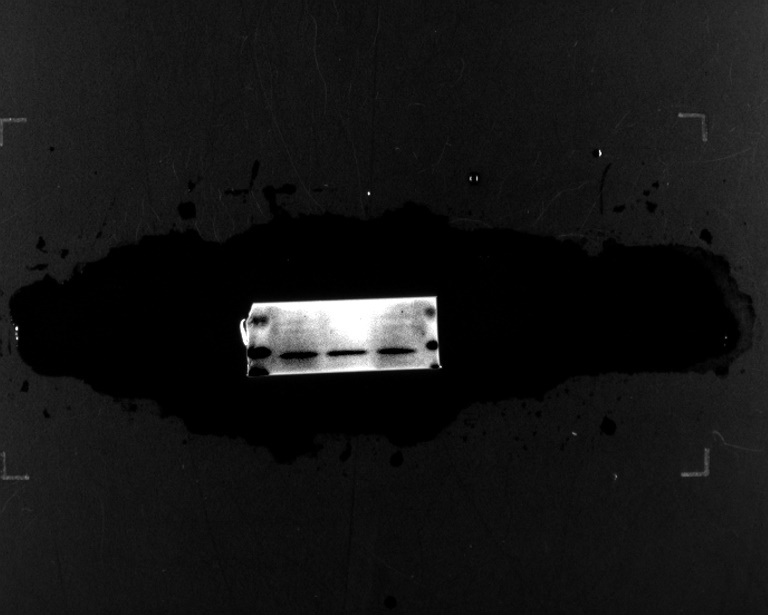

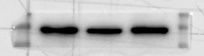


GAPDH
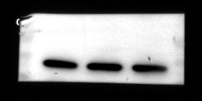

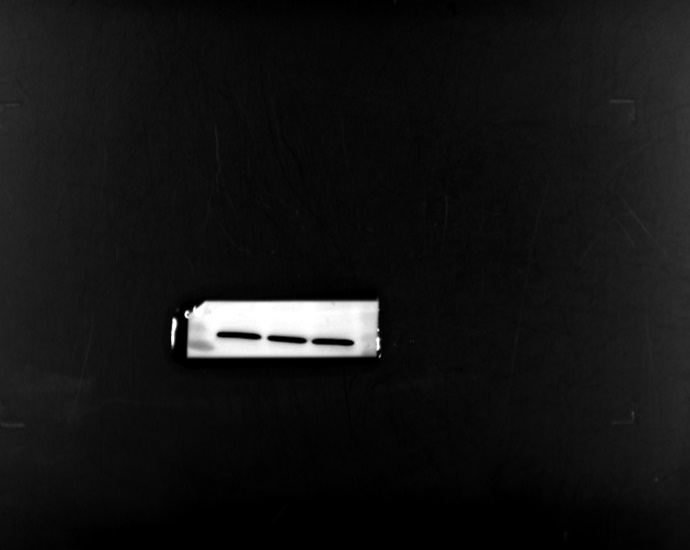

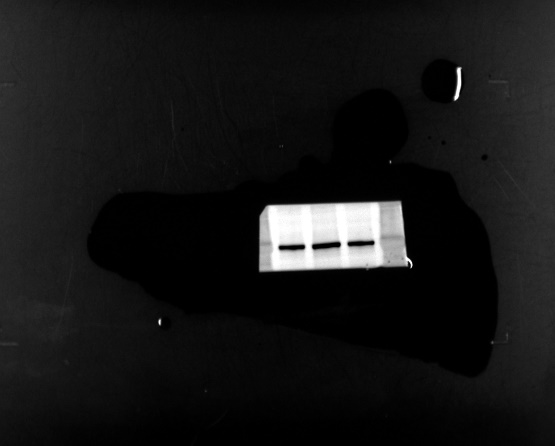


IB AKT
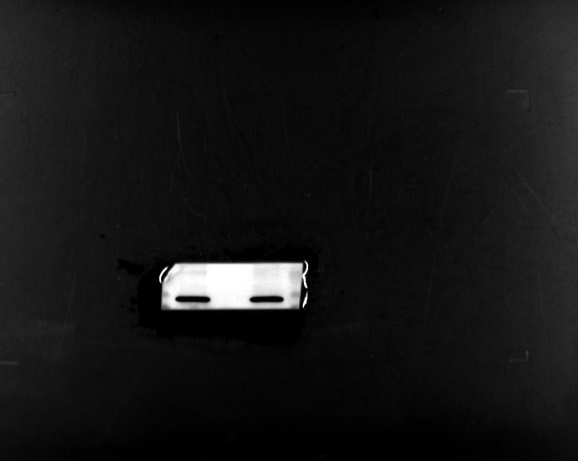

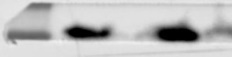


IB KIF14
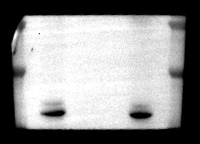

Supplement: Supplementary file 1 — Supplementary Material [file j_biol-2025-1324_suppl_001.docx]
